# The value of rapid functional assays of germline *p53* status in LFS and LFL families

**DOI:** 10.1054/bjoc.1999.1054

**Published:** 2000-02-21

**Authors:** R S Camplejohn, N Sodha, R Gilchrist, M E Lomax, P M Duddy, C Miner, P Alarcon-Gonzalez, D M Barnes, R A Eeles

**Affiliations:** Richard Dimbleby Department of Cancer Research, Guy's, Kings and St Thomas' Medical School, St Thomas' Hospital, London, SE1 7EH, UK; The Cancer Genetics Team, Institute of Cancer Research and Royal Marsden Hospital Trust, Fulham Road, London, SW3 6JJ, UK; ICRF Breast Pathology Unit, Guy's Hospital, London, SE1 9RT, UK; University of Valladolid, Spain

**Keywords:** p53, Li–Fraumeni syndrome, apoptosis, FASAY

## Abstract

We have tested two rapid assays of p53 function, namely the apoptotic assay and the FASAY as means of detecting germline *p53* mutations in members of Li–Fraumeni and Li–Fraumeni-like families. Results of the functional assays have been compared with direct sequencing of all 11 exons of the *p53* gene. The results show good agreement between the two functional assays and between them and sequencing. No false-positives or negatives were seen with either functional assay although the apoptotic assay gave one borderline result for an individual without a mutation. As an initial screen the apoptotic assay is not only rapid but inexpensive and very simple to perform. It would be expected to detect any germline defect that leads to loss of p53 function. The apoptotic assay could be ideal as a means of prescreening large numbers of samples and identifying those that require further investigation. The FASAY detects mutations in exons 4–10, is rapid and distinguishes between functionally important and silent mutations. © 2000 Cancer Research Campaign


*British Journal of Cancer* (2000) **82**(6), 1145–1148
© 2000 Cancer Research Campaign
Article no. bjoc.1999.1054, available online at http://www.idealibrary.com on IDEAL®

### The value of rapid functional assays of germline *p53* status in LFS and LFL families

**RS Camplejohn[1], N Sodha[2], R Gilchrist[1], ME Lomax[1], PM Duddy[1], C Miner[4], P Alarcon-Gonzalez[4], DM Barnes[3] and RA Eeles[2]**

[1]Richard Dimbleby Department of Cancer Research, Guy's, Kings and St Thomas' Medical School, St Thomas' Hospital, London SE1 7EH, UK; [2]The Cancer Genetics Team, Institute of Cancer Research and Royal Marsden Hospital Trust, Fulham Road, London SW3 6JJ, UK; [3]ICRF Breast Pathology Unit, Guy's Hospital, London SE1 9RT, UK; [4]University of Valladolid, Spain

**Summary** We have tested two rapid assays of p53 function, namely the apoptotic assay and the FASAY as means of detecting germline *p53* mutations in members of Li–Fraumeni and Li–Fraumeni-like families. Results of the functional assays have been compared with direct sequencing of all 11 exons of the *p53* gene. The results show good agreement between the two functional assays and between them and sequencing. No false-positives or negatives were seen with either functional assay although the apoptotic assay gave one borderline result for an individual without a mutation. As an initial screen the apoptotic assay is not only rapid but inexpensive and very simple to perform. It would be expected to detect any germline defect that leads to loss of p53 function. The apoptotic assay could be ideal as a means of prescreening large numbers of samples and identifying those that require further investigation. The FASAY detects mutations in exons 4–10, is rapid and distinguishes between functionally important and silent mutations. © 2000 Cancer Research Campaign

**Keywords:** p53; Li–Fraumeni syndrome; apoptosis; FASAY

Rare cancer-prone families were first recognized in the late 1960s with a very particular clinical picture involving early onset sarcomas, breast carcinoma, brain tumours, leukaemias and adrenal carcinoma (Li and Fraumeni 1969); these families are said to exhibit Li–Fraumeni syndrome (LFS). More recently, families have been recognized with similar spectra of malignancies, which do not fully meet the precise criteria of classical LFS (Birch et al, 1994; Eeles, 1995). Such families have been called Li–Fraumeni-like (LFL). In 1990, Malkin et al demonstrated a relationship between germline *p53* mutations and LFS. *p53* is a tumour suppressor gene and mutation and deletion of the gene are the most common genetic defects seen in sporadic clinical cancer. Recent reports indicate that up to 75% of families with classical LFS have germline mutations in the *p53* gene as do 10–22% of LFL families (Eeles, 1995; Varley et al, 1997). The identification of these mutations is important clinically because carriers are at markedly increased risk of developing cancer (cumulative lifetime risk in women 90%) and may be at greater risk of radiation-induced carcinogenesis (Eeles, 1995).

Point mutations, which disrupt p53 function, were originally thought to be largely restricted to the core DNA binding region of *p53*. However, this central area of *p53* has been more extensively investigated for mutations than either the N- or C-termini, therefore the frequency of mutations outside the core domain may be higher than is thought (Casey et al, 1996). Thus, if sequencing is to be used to detect all coding germline *p53* mutations, then all exons and splice junctions should be investigated (Varley et al, 1997). The families with p53-related germline defects are rare and they often first come to light due to a high incidence of cancer. A rapid screening assay would be valuable to determine, from the much larger number of cancer-prone families, the minority due to a p53-related defect. We have tested two potential assays of p53 function on a number of LFS/LFL family members as part of a larger study. The first of these assays was developed in our laboratory and depends upon the measurement of the apoptotic response of peripheral blood lymphocytes (PBL) to radiation-induced DNA damage (Camplejohn et al, 1995). In individuals with functional p53 most PBL die within 48 h of a dose of 4 Gy radiation, whilst in individuals with an inherited defect leading to loss of p53 function PBL are resistant to the lethal effects of radiation. We believe that this assay will detect any inherited p53 defect which results in loss of p53 function. The second test, the FASAY, is a yeast-based assay which tests the ability of p53 protein to transcriptionally transactivate a target gene by binding to the RGC consensus sequence (Flaman et al, 1995; Lomax et al, 1997). Functional p53 leads to the growth of white yeast colonies, whilst mutant p53 results in red colonies. This assay is also rapid, inexpensive and links any detected defect directly to exons 4–10 of the *p53* gene.

*Received 3 March 1999*
*Revised 2 September 1999*
*Accepted 2 November 1999*

*Correspondence to:* RS Camplejohn

### MATERIALS AND METHODS

### Subjects

A minimum of 20 ml of blood was collected from 20 affected and unaffected members of LFS/LFL families and from 50 normal individuals with no history of excessive cancer occurrence in their families. Data on all LFS/LFL family members for whom samples were received between the beginning of 1994 and the middle of 1997 are included in this study provided that results for all three assay methods are available.

### Apoptotic assay

PBL were separated from fresh whole blood samples and cultured in standard medium (RPMI, 10% fetal calf serum, glutamine and antibiotics) for three days. At 72 h of culture, half of the cells were exposed to 4 Gy of gamma radiation. Both irradiated and control cells were returned to the incubator for a further 24 h at which point the cells were fixed in 70% ethanol at 4°C. For analysis, the samples were acid-denatured for 12 min in 0.1 M hydrochloric acid at 37°C and then stained with propidium iodide. The amount of apoptosis was assessed by the size of the sub-G1 peak on DNA profiles. In all but a few cases where there were insufficient cells, flow cytometry was performed in triplicate with good reproducibility. This flow cytometric method has been validated by comparison with other techniques, including electron microscopic counting and cell sorting of apoptotic cells (Camplejohn et al, 1995) to confirm that the sub-G1 peak did consist of apoptotic cells.

### FASAY

mRNA extracted from phytohaemagglutinin (PHA)-stimulated PBL was subjected to the FASAY (Flaman et al, 1995; Lomax et al, 1997). In this assay, purified mRNA was subjected to reverse transcription polymerase chain reaction (RT-PCR). The unpurified *p53* RT-PCR product was then co-transfected into yeast with a linearized expression vector carrying the 5′ and 3′ ends of the *p53* open reading frame. This results in the constitutive expression of the human p53 protein present. Yeast that have repaired the plasmid were selected by their ability to grow in the absence of leucine and with a limiting concentration of adenine; if the *p53* is wild-type the *ADE2* gene is activated, producing adenine and white colonies are formed. Mutant *p53* does not activate the *ADE2* gene and the resultant colonies are red due to the accumulation of a red adenine precursor. This assay recognizes mutations from exon 4 to exon 10 of the *p53* gene as this is the extent of the PCR product that recombines with the gapped vector. Many control and repeat experiments were performed to demonstrate the reproducibility of the FASAY, some of these are described in Lomax et al (1997).

### Sequencing

DNA was isolated from PBL by the sucrose lysis method. DNA fragments were PCR-amplified using the primer sequence below:

| | | | |
|---|---|---|---|
| Exon 1 | – | Forward | 5′-GAGAATCCTGACTCTGCACC |
| | | Reverse | 5′-AGCCGAGCCCGTGACTCA |
| Exons 2/3 | – | Forward | 5′-ATGCTGGATCCCCACTTTTC |
| | | Reverse | 5′-AAGAGCAGTCAGAGGACCAG |
| Exon 4 | – | Forward | 5′-GACCTGGTCCTCTGACTGCT |
| | | Reverse | 5′-GCATTGAAGTCTCATGGAAG |
| Exon 5 | – | Forward | 5′-ACTTGTGCCCTGACTTTCAACT |
| | | Reverse | 5′-CAATCAGTGAGGAATCAGAGGC |
| Exon 6 | – | Forward | 5′-TCAGATAGCGATGGTGAGCAG |
| | | Reverse | 5′-GCCACTGACAACCACCCTTA |
| Exon 7 | – | Forward | 5′-AGGCGCACTGGCCTCATCTT |
| | | Reverse | 5′-GAAATCGGTAAGAGGTGGGC |
| Exon 8 | – | Forward | 5′-GGAGTAGATGGAGCCTGGTTT |
| | | Reverse | 5′-GGTGATAAAAGTGAATCTGAGGC |
| Exon 9 | – | Forward | 5′-GGAGACCAAGGGTGCAGGTTAT |
| | | Reverse | 5′-GTTAGTTAGCTACAACCAGGAGCC |
| Exon 10 | – | Forward | 5′-CAATTGTAACTTGAACCATC |
| | | Reverse | 5′-ATGAGAATGGAATCCTATGG |
| Exon 11 | – | Forward | 5′-CTCACTCATGTGATGTCATC |
| | | Reverse | 5′-CAAAATGGCAGGGGAGG. |

The annealing temperature was 56°C for exons 2–3, 4, 10 and 11 and 60°C for exons 1 and 5–9. To remove the excess primers and dNTPs before sequencing, the PCR products were purified by ammonium acetate and isopropanol precipitation. Cycle-sequencing of both strands was performed using either of the two primers used in PCR with dye-terminator chemistry and AmplitaqFS (Perkin-Elmer Applied Biosystems). To remove excess dye before loading samples on to the sequencer the samples were purified by sodium acetate and ethanol precipitation. The products were analysed on an ABI 310 sequencer.

### RESULTS

In this report the results of the apoptotic assay and the FASAY are compared with each other and with the results of sequencing. Data from 20 affected and unaffected individuals who are members of LFS/LFL families are included (Table 1). These data are compared to results from 50 normal individuals with no known inherited susceptibility to cancer. This paper includes data on LFS/LFL family members collected up to the middle of 1997 as part of an on-going larger study in which sporadic cancer patients and members of other types of cancer-prone family (e.g. BRCA1 and BRCA2) were also investigated. From this larger database we have defined a normal range of values for the apoptotic assay and a range denoting the likely presence of a heterozygous germline *p53* mutation. A normal apoptotic response is thus defined as anything greater than 27%, whilst any value below 23% is consistent with the presence of a mutation. This leaves a borderline region of 23–27% into which occasional results fall. Such results are considered as suspicious and worthy of further investigation, initially with the FASAY.

When comparing the various assays, the results show excellent agreement between the two functional assays and between them and sequencing. Both the FASAY and the apoptotic assay successfully detected all known carriers of germline *p53* defects. In the apoptotic assay these carriers had responses significantly lower than those seen in normal individuals and non-affected members of LFS/LFL families (see Figure 1). Further, in the FASAY all carriers of germline *p53* mutations yielded between 44 and 60% red colonies, well above the maximum background level of 10%. In addition, no individuals from the LFS/LFL families who had homozygous wild-type *p53* were classified as abnormal by either functional assay, though one such individual did yield a borderline apoptotic assay result.

### DISCUSSION

In this study, both of the functional assays detected all known carriers of germline *p53* mutations. Although it requires to be established with a larger series of mutation carriers, we would

*© 2000 Cancer Research Campaign*

**Table 1** Apoptotic assay results denote a mutation (Mut) if the increase in apoptotic cells after 4 Gy radiation is < 23%, borderline (B) [23–27%] and wild-type (WT) [> 27%]

| Case | Family status | Cancer diagnosed in individual | Apoptotic assay | FASAY | Sequencing of exons 1–11 |
|---|---|---|---|---|---|
| 4001 | LFL | Yes | Mut | Mut | Mut codon 151 |
| 4002 | LFS | Yes | Mut | Mut | Mut codon 248 (subject II-2 family 266, Varley et al 1997) |
| 4005 | LFL | Yes | Mut | Mut | Mut codon 337 |
| 4006 | LFL | Yes | Mut | Mut | Mut codon 245 (subject F, Camplejohn et al 1995) |
| 5020 | LFL | Yes | Mut | Mut | Mut codon 273 |
| 6034 | LFS | Yes | Mut | Mut | Mut codon 213 |
| 5004 | LFL | No | WT | WT | WT |
| 5006 | LFL | No | WT | WT | WT |
| 5010 | LFL | No | WT | WT | WT |
| 6023 | LFS | No | WT | WT | WT |
| 6024 | LFS | No | WT | WT | WT |
| 6033 | LFL | No | WT | WT | WT |
| 6072 | LFL | No | WT | WT | WT |
| 6107 | LFL | No | WT | WT | WT |
| 6135 | LFL | No | WT | WT | WT |
| 6137 | LFL | No | B | WT | WT |
| 6141 | LFL | No | WT | WT | WT |
| 6146 | LFL | No | WT | WT | WT |
| 7094 | LFL | No | WT | WT | WT |
| 7099 | LFS | No | WT | WT | WT |

These figures are based on a study of around 300 samples. Results on 50 normal volunteers yielded a mean apoptotic response of 44% (1 s.d. 9%). The FASAY was performed as described by Flaman et al (1995) and Lomax et al (1997); 10% or less red colonies denotes a homozygous wild-type result, > 10% red colonies denotes the presence of a mutation.

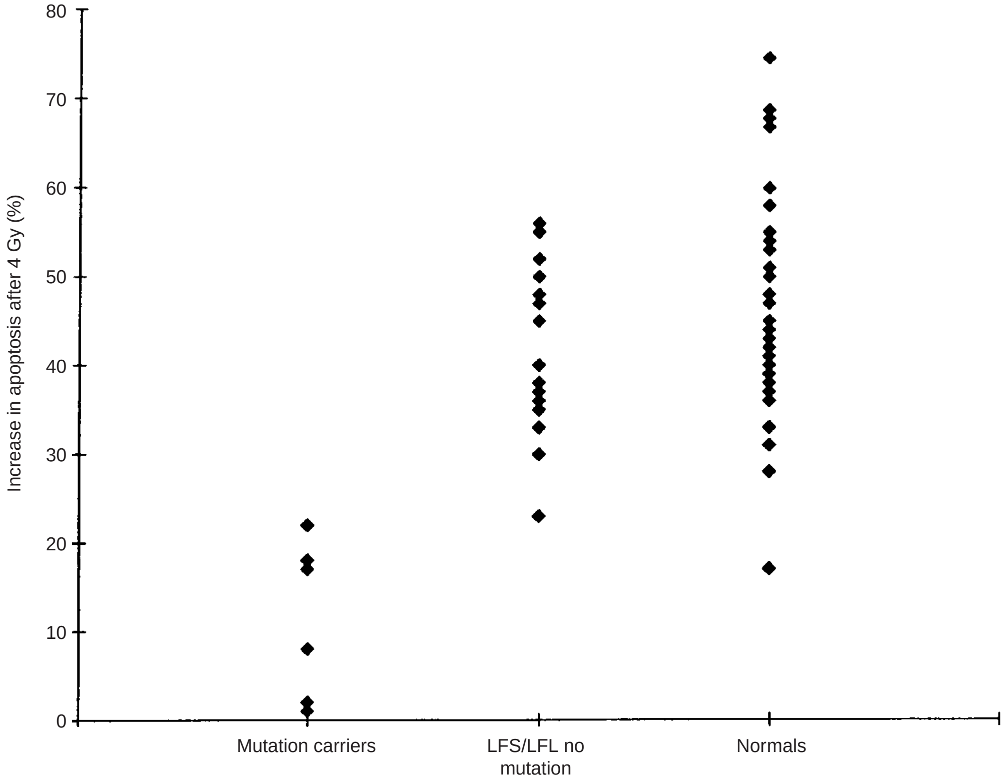

**Figure 1** The figure shows the distribution of values for the apoptotic response in groups of LFS/LFL individuals with and without *p53* mutations and normal volunteers. The mutation group differs significantly from the LFS/LFL group lacking a mutation ($P = 3 \times 10^{-5}$) and the normal group ($P = 3 \times 10^{-7}$). The unaffected and control groups do not differ significantly ($P = 0.4$). Statistical comparison was done using Fisher's exact test. Only one sample from the unaffected and control groups has a lower apoptotic response than any of the affected individuals. Unfortunately, this individual was no longer available for further study

*© 2000 Cancer Research Campaign*

expect, in principle, that all germline defects which lead to loss of p53 function would be detected by the apoptotic assay. However, the FASAY will only detect mutations between exons 4 and 10 and will miss mutations which lead to loss of expression of one allele. In addition, we have found that a splice donor mutation in exon 4, which leads to the production of three species of aberrant mRNA was not detected by the FASAY (Varley et al, 1998). We also have evidence from other studies on sporadic breast tumours that the FASAY may fail to detect at least some stop mutations in the *p53* gene (RS Camplejohn, unpublished data). However, in the current study as well as detecting all carriers of germline *p53* mutations, no individuals with two wild-type alleles were wrongly identified as carriers of inherited defects; in the FASAY all such individuals had < 10% red colonies. In the apoptotic assay non-affected individuals all gave results above the maximum level seen in gene carriers, apart from one who gave a borderline result (see Table 1 legend for details). A larger study of sporadic cancer patients and members of families with other inherited cancer predispositions suggests that such results are seen occasionally for reasons that are not yet clear. In addition, a sample from one normal volunteer gave a low apoptotic response in the range seen with family members having a *p53* mutation. The probability of this individual having a germline *p53* mutation is very low but, unfortunately, no material was available for further study. However, the apoptotic assay would only be used as an initial screening technique to identify cases worthy of further investigation by more detailed methods such as sequencing. Thus a small number of borderline, or even false-positive, results would not be a major problem in the event of the assay being used as a clinical screening test. Therefore, we consider that both assays show real promise as a screening process to identify samples which warrant further molecular sequencing analysis and both have a potential role in identifying germline *p53* mutations in members of cancer-prone families. As an initial screen the apoptotic assay does have the great advantages of simplicity and low cost.

### ACKNOWLEDGEMENTS

We would like to thank Dr G Turner, Leeds, Dr K MacDermot, Royal Free Hospital, London, Mrs A Ardern-Jones, Dr R Houlston and Dr V Murday Royal Marsden Hospital Trust for supplying some of the blood samples from which the above data were obtained. This work was supported in part by grants from the Medical Research Council and the Special Trustees for St Thomas's Hospital.

### REFERENCES

Birch JM, Hartley AL, Tricker KJ, Prosser J, et al (1994) Prevalence and diversity of constitutional mutations in the p53 gene among 21 Li–Fraumeni families. *Cancer Res* **54**: 1298–1304

Camplejohn RS, Perry P, Hodgson SV, Turner G, et al (1995) A possible screening test for inherited p53-related defects based on the apoptotic response of peripheral blood lymphocytes to DNA damage. *Br J Cancer* **72**: 654–662

Casey G, Lopez ME, Ramos JC, Plummer SJ, et al (1996) DNA sequence analysis of exons 2 through 11 and immunohistochemical staining are required to detect all known p53 alterations in human malignancies. *Oncogene* **13**: 1971–1981

Eeles RA (1995) Germline mutations in the TP53 gene. *Cancer Surv* **25**: 101–124

Flaman JM, Frebourg T, Moreau V, Charbonnier F, et al (1995) A simple p53 functional assay for screening cell lines, blood and tumours. *Proc Natl Acad Sci USA* **92**: 3963–3967

Li FP and Fraumeni JF (1969) Soft-tissue sarcomas, breast cancer and other neoplasms. *Ann Int Med* **71**: 747–752

Lomax ME, Barnes DM, Gilchrist R, Picksley SM, Varley JM and Camplejohn RS (1997) Two functional assays employed to detect an unusual mutation in the oligomerisation domain of p53 in a Li–Fraumeni like family. *Oncogene* **14**: 1869–1874

Malkin D, Li FP, Strong LC, Fraumeni JF, et al (1990) Germline p53 mutations in a familial syndrome of breast cancer, sarcomas and other neoplasms. *Science* **250**: 1233–1238

Varley JM, Mcgown G, Thorncroft M, Santibanezkoref MF, Kelsey AM, Tricker KJ, Evans DGR and Birch JM (1997) Germ-line mutations of TP53 in Li–Fraumeni families: an extended study of 39 families. *Cancer Res* **57**: 3245–3252

Varley JM, Chapman P, Mcgown G, Thorncroft M, et al (1998) Genetic and functional studies of a germline TP53 splicing mutation in a Li–Fraumeni-like family. *Oncogene* **16**: 3291–3298

*© 2000 Cancer Research Campaign*
